# Comparison of Diabetes Risk Score Estimates and Cardiometabolic Risk Profiles in a Middle-Aged Irish Population

**DOI:** 10.1371/journal.pone.0078950

**Published:** 2013-11-13

**Authors:** Catherine M. Phillips, Patricia M. Kearney, Vera J. McCarthy, Janas M. Harrington, Anthony P. Fitzgerald, Ivan J. Perry

**Affiliations:** 1 HRB Centre for Diet and Health Research, Dept. of Epidemiology and Public Health, University College Cork, Cork, Ireland; 2 Dept. of Statistics, University College Cork, Cork, Ireland; Imperial College London, United Kingdom

## Abstract

**Background:**

To compare diabetes risk assessment tools in estimating risk of developing type 2 diabetes (T2DM) and to evaluate cardiometabolic risk profiles in a middle-aged Irish population.

**Methods:**

Future risk of developing T2DM was estimated using 7 risk scores, including clinical measures with or without anthropometric, biological and lifestyle data, in the cross-sectional Mitchelstown cohort of 2,047 middle-aged men and women. Cardiometabolic phenotypes including markers of glucose metabolism, inflammatory and lipid profiles were determined.

**Results:**

Estimates of subjects at risk for developing T2DM varied considerably according to the risk assessment tool used (0.3% to 20%), with higher proportions of males at risk (0–29.2% vs. 0.1–13.4%, for men and women, respectively). Extrapolated to the Irish population of similar age, the overall number of adults at high risk of developing T2DM ranges from 3,378 to 236,632. Numbers of non-optimal metabolic features were generally greater among those at high risk of developing T2DM. However, cardiometabolic profile characterisation revealed that only those classified at high risk by the Griffin (UK Cambridge) score displayed a more pro-inflammatory, obese, hypertensive, dysglycaemic and insulin resistant metabolic phenotype.

**Conclusions:**

Most diabetes risk scores examined offer limited ability to identify subjects with metabolic abnormalities and at risk of developing T2DM. Our results highlight the need to validate diabetes risk scoring tools for each population studied and the potential for developing an Irish diabetes risk score, which may help to promote self awareness and identify high risk individuals and diabetes hot spots for targeted public health interventions.

## Introduction

Type 2 diabetes mellitus (T2DM) is a common metabolic condition associated with increased morbidity and mortality, largely due to increased cardiovascular risk [1]–[3]. The increasing global prevalence of T2DM represents a major public health concern. Current estimates predict in excess of 400 million individuals with T2DM worldwide by 2030 [4]. The diabetes epidemic has been driven by complex gene-environment interactions. Genetic factors contribute almost 50% towards T2DM risk. Obesity and weight gain are also directly related to T2DM risk [5]. The pathway from obesity, impaired fasting glucose (IFG) and insulin resistance towards impaired glucose tolerance (IGT) and overt T2DM represents a progressive phenotype. T2DM is preventable through lifestyle changes in diet and physical activity [6], [7], and subjects with IFG and IGT have been a key focus of prevention studies [6], [8], [9]. However population screening for IGT is time consuming and cost prohibitive. Moreover longitudinal studies have shown that only about half of subjects with IFG and/or IGT progress to T2DM and approximately 40% of subjects who developed T2DM had normal glucose tolerance at baseline [10]. Thus developing more cost-effective, simple and fast population applicable screening methods to identify those at risk and who might benefit from targeted prevention is a current challenge.

Various diabetes risk assessment tools have been developed in numerous populations, either for self-assessment relying on readily available health information; others need to be completed by the physician and require clinical and/or biological data [11]–[16]. Using a variety of risk factors, weighting schemes and thresholds such risk scores aim to identify those with prevalent but undiagnosed diabetes and/or incident diabetes. Considering the long asymptomatic period preceding the manifestation of T2DM, early identification of individuals at increased risk could allow earlier diagnosis, enabling earlier targeted interventions such as implementation of healthy lifestyle changes in nutritional behaviour and exercise or pharmacotherapy, thus attenuating development of diabetes and its associated cardiometabolic complications. The prevalence of T2DM in an Irish primary care based sample in 1998 was estimated to be 3.9%; 30% of whom were undiagnosed [17]. A recent report from the Irish Institute of Public Health suggested a prevalence of 8.9% and predicted a 30% increase over the next decade [18]. We recently examined the prevalence of diagnosed and undiagnosed diabetes within the Mitchelstown cohort [19]. Estimates of 8.5% were comparable to that from the nationally representative general population [20]. However a considerable proportion (41%) of diabetes cases were undiagnosed, emphasising the need for more effective detection strategies. No diabetes risk scores have been applied to or developed in an Irish population where diabetes care represents approximately 10% of the Republic of Irelands total health expenditure [21]. Therefore the primary aims of this study were to compare the results of diabetes risk scores based on a range of anthropometric, clinical, biological, family history and/or lifestyle data in estimating risk of developing T2DM in a middle-aged cohort and to characterise cardiometabolic profiles according to each tool. Secondary objectives included extrapolation of these findings to the Irish population of same age and gender, and assessment of the impact of different diagnostic criteria to exclude T2DM (fasting plasma glucose (FPG) and haemoglobin A_1c_ (HbA_1c_) on estimated diabetes risk.

## Methods

### Study Design and Population

The Cork and Kerry Diabetes and Heart Disease Study (Phase II) was a single centre, cross-sectional study conducted between 2010 and 2011 [22]. A population representative random sample was recruited from a large primary care centre (Livinghealth Clinic) in Mitchelstown, County Cork, Ireland, which includes 8 general practitioners and serves a catchment area of approximately 20,000 with a mix of urban and rural residents. Mitchelstown cohort participants were randomly selected from all registered attending patients in the 50–69 year age group. In total 3,807 potential participants were selected from the practice list. Following exclusion of duplicates, deaths and ineligibles, 3,043 were invited to participate in the study and of these 2,047 (49.2% male) completed the questionnaire and physical examination components of the baseline assessment (response rate 67%). While almost 100% of the cohort were in the 50–69 year age bracket it should be noted that a small number of subjects outside of this age group, mainly subjects who celebrated their 70^th^ birthday during the cohort recruitment, were included. Ethics committee approval conforming to the Declaration of Helsinki was obtained from the Clinical Research Ethics Committee of University College Cork. All participants provided written informed consent. Individuals with doctor diagnosed diabetes or FPG ≥7.0 mmol/L were excluded, resulting in 1,862 non-diabetic individuals at risk of developing T2DM who were included in the current study. For comparative purposes HbA_1c_ was also used to define T2DM (HbA_1c_ ≥6.5% and/or treatment for diabetes), resulting in 1,823 non-diabetic individuals for analyses. Participants with missing data for FPG, HbA_1c_ or diabetes treatment were excluded. All collected source data are maintained and stored at the study research office, in the Department of Epidemiology and Public Health, University College Cork. Specific proposals for future collaboration for which data would be made available would be welcomed. Further information can be found on the Centre for Diet and Health Research website, http://www.ucc.ie/en/hrbc/projects/cluster3 or by contacting the corresponding author.

### Clinical and Anthropometric Data

All participants attended the clinic in the morning after an overnight fast (minimum 8 h). Fasting blood samples were taken on arrival. Participants completed a General Health Questionnaire (GHQ), a Food Frequency Questionnaire (FFQ) and the International Physical Activity Questionnaire (IPAQ). Data on age, gender, family history, medication/medical history and lifestyle factors was gathered through a self-completed GHQ. Participants answered questions regards personal and family diabetes diagnosis/treatment and personal hypertension diagnosis/treatment. Corticosteroid use was also used in the current analysis. Smoking status was defined as never, former and current smokers. Alcohol consumption included questions regards past and current intake to define drinkers, never or former drinkers. Diet was assessed using a modified version of the EPIC FFQ, validated for use in the Irish population, which was previously used in the Cork and Kerry Phase 1 study [23]. Participants were classified according to number of daily portions of fruit and vegetable, red meat (150 g/day), wholegrain bread (50 g/day), coffee (150 g/day) and moderate alcohol consumption (10–40 g/day). Physical activity levels were assessed using the short form IPAQ [24]. Subjects were defined as having low, moderate or high levels of physical activity. Blood pressure and resting pulse were measured according to the European Society of Hypertension Guidelines using an Omron M7 Digital BP monitor on the right arm, after a 5 minute rest in the seated position. The average of the second and third measurements was used for analyses. Anthropometric measurements were recorded with calibrated instruments according to a standardised protocol. Body weight was measured in kilograms without shoes, to the nearest 100 g, using a Tanita WB100MA weighing scales (Tanita Corporation, IL, USA). Height was measured in centimetres to 1 decimal place using a Seca Leicester height gauge (Seca, Birmingham, UK). Body mass index (BMI) was calculated. Hip circumference at widest point and mid-way waist circumference were measured in centimetres to 1 decimal place using a Seca 200 measuring tape (Seca, Birmingham, UK). The average of two measures was used for analyses.

### Biological Analyses

Plasma and serum were prepared from fasting blood samples. FPG concentrations were determined using a glucose hexokinase assay and serum high density lipoprotein (HDL) cholesterol, low density lipoprotein cholesterol, triglycerides and uric acid levels were analysed using enzymatic colorimetric tests (Olympus Life and Material Science Europa Ltd., Lismeehan, Co. Clare, Ireland) on an Olympus 5400 automatic analyser (Olympus Diagnostica Gmbh, Hamburg, Germany) by Cork University Hospital Biochemistry Laboratory. HbA_1c_ was measured using an automated high-performance liquid chromatography analyser (Tosoh HLC-723 (G7), Tosoh Europe N.V, Tessenderlo, Belgium). Complement component c3 (C3) was determined by immunoturbidimetric assay (Rx Daytona; Randox Laboratories, Antrim, UK). Serum insulin, C reactive protein (CRP), tumour necrosis factors aTNF-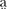 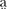
interleukin 6 (IL-6), adiponectin (ACDC), leptin and plasminogen activator inhibitor-1 (PAI-1)were determined using a biochip array system (Evidence Investigator; Randox Laboratories, Antrim, UK). Homeostasis model assessment (HOMA), a measure of insulin resistance, was calculated as [(fasting plasma glucose x fasting serum insulin)/22.5] [25].

### Diabetes Risk Scores

Seven diabetes risk scores were assessed including the 9-year risk score based on the French DESIR study by Balkau *et al.*, [11], the UK Cambridge Diabetes Risk Score by Griffin *et al.*, [12], the 10-year basic risk score, based on lifestyle and clinical information, and the enhanced risk score which incorporates biological factors from Kahn *et al.*, [13], the 5–10 year Finnish Diabetes Risk Score (FINDRISC) [14], the 5-year German Diabetes Risk Score by Schulze *et al.*, [15] and finally the 8-year risk score from Wilson *et al.*, based on the Framingham Offspring Study [16]. Further details regarding the study populations and variables used in each risk score are presented in Tables S1 and S2.

### Statistical Analysis

Proportions of individuals at high risk of developing T2DM were calculated according to each diabetes risk score and expressed as percentages and 95% confidence intervals (CI). Cardiometabolic and inflammatory markers were assessed for normality of distribution, and skewed variables were normalised by log_10_ transformation as appropriate. Differences between groups were analyzed by independent t-tests or Mann Whitney U tests. Non-optimal cardiometabolic risk features were determined using the NCEP ATP III metabolic syndrome (MetS) criteria [26]. Standard BMI and 75^th^ percentile cut-offs were used for HOMA and for generating a composite inflammatory score based on C3, CRP, TNF-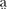
IL-6, ACDC and leptin concentrations. The combined number of each of these non-optimal features according to each score was then compared between individuals classified as at high risk of developing T2DM and those classified as not being at high risk by independent t-tests. For the extrapolation analysis we used current national age-group specific diabetes prevalence estimates [18] to the population estimates for 2011 provided by the Central Statistics Office (www.cso.ie) to ascertain the number of non-diabetic subjects in Ireland. Next the number of at risk subjects was estimated for each risk score by applying the age-group and gender-specific estimates obtained in the Mitchelstown cohort to the corresponding diabetes-free population. Correlations and pair-wise comparison of agreement between risk scores were assessed by Spearman correlation coefficient and Cohen’s kappa, respectively. Statistical analyses were carried out using SPSS version 18.0 for Windows (SPSS Inc, Chicago, IL. USA).

## Results

### Estimated Proportion of Mitchelstown Participants at High Risk for T2DM

**Figure 1:**
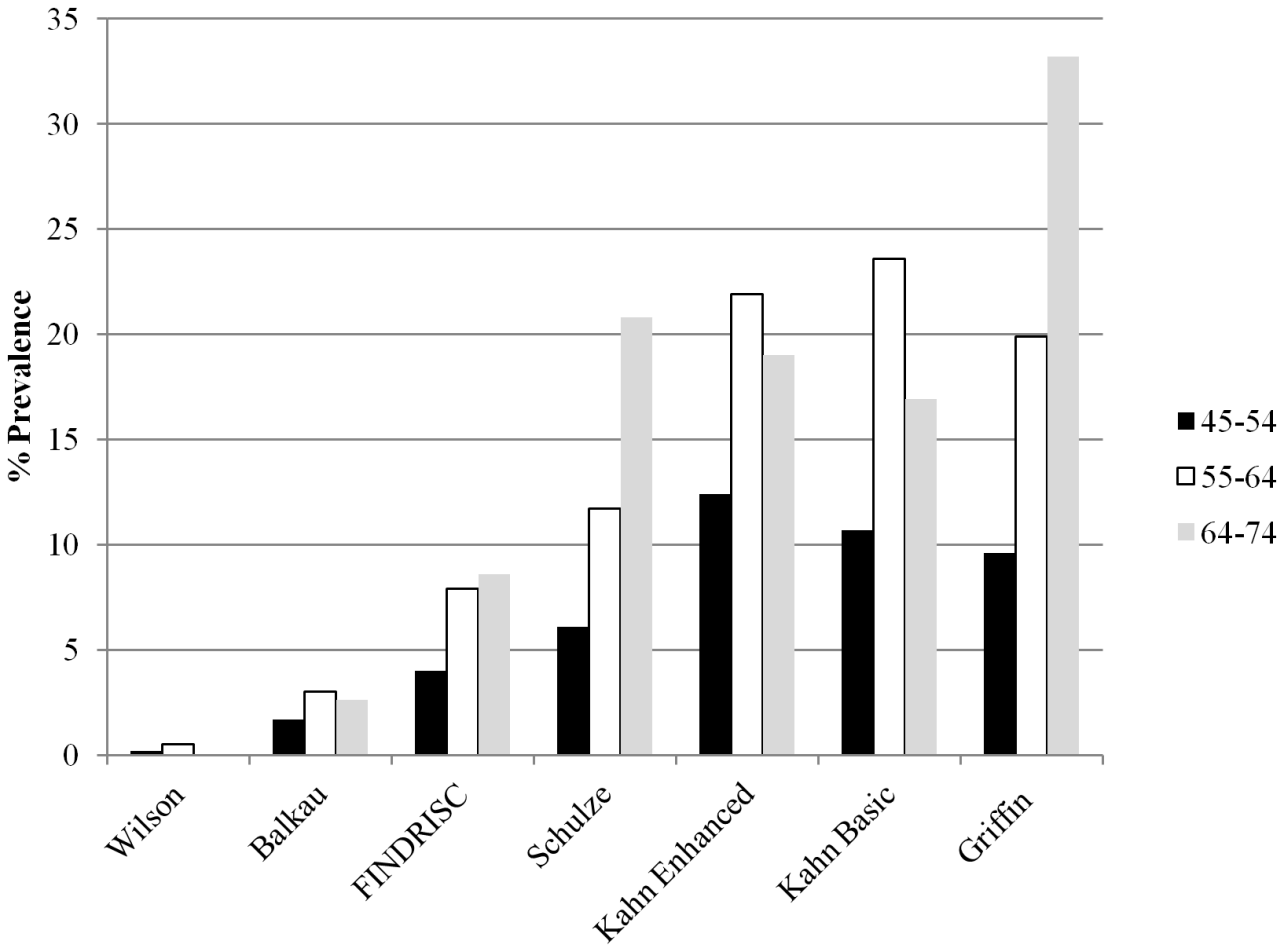
Comparison of the proportion of Mitchelstown cohort subjects at risk of developing T2DM according to each diabetes risk score and age group. Lowest risk was identified in the 45–54 year old group (black bars) for every diabetes risk score. Greatest risk was detected in the 55–64 year olds (white bars) for the Balkau and both Kahn risk scores, whereas the FINDRISC, Schulze and Griffin risk scores demonstrated greatest risk in the 65–74 year old individuals (grey bars).

Characteristics of the non-diabetic individuals (defined by FPG) included in the analyses are presented in Table S3. Following exclusion of missing data and existing T2DM the remaining 1,862 and 1,823 non-diabetic participants (by FPG and HbA_1c_ cut-offs, respectively) were used in the analyses. The proportion of individuals in the Mitchelstown cohort at high risk for developing T2DM is presented in Table 1. Estimates varied considerably between scores (from 0.3% [Wilson] to 20% [Griffin]). Higher risk was detected in men relative to women with the exception of the Balkau risk score, which did not identify any males at increased risk of developing T2DM. Choice of diagnostic criteria to exclude diabetes did not significantly impact on estimated incidence. Similar but slightly lower numbers of high risk males and females were identified using the Wilson, Balkau, FINDRISC, Schulze and Kahn Basic scores when HbA_1c_ was used to exclude T2DM. In contrast higher proportions of at risk subjects were identified using the Kahn Enhanced score, whereas identical percentage values were obtained using the Griffin score. Examination of estimated risk according to age group across each risk score consistently showed lowest risk in the 45–54 year age group (Figure 1). Greatest risk was identified in the 65–74 year olds according to the FINDRISC, Schulze and Griffin risk scores, whereas the Balkau and both Kahn risk scores detected highest risk in the 55–64 year olds. Extrapolated to the Irish population of similar age (Table 2), the overall number of adults at high risk of developing T2DM ranges from 3,378 to 236,632. Exclusion of the lowest scores (Wilson and Balkau) yields higher estimates ranging from 80,381 to 233,431.

**Table 1:** Proportion (percentage and 95% confidence interval) of subjects at risk of developing T2DM in the Mitchelstown cohort according to each diabetes risk score by gender using FPG and HbA_1c_ to exclude existing diabetes.

	N	Wilson	Balkau	FINDRISC	Schulze	Kahn Enhanced	Kahn Basic	Griffin
*FPG*								
All	1862	0.3 (0.06–0.58)	2.6 (1.9–3.3)	7.0 (5.9–8.2)	12.1 (10.6–13.6)	18.9 (17.1–20.6)	18.9 (17.1–20.7)	20.0 (18.2–21.9)
Male	896	0.6 (0.07–1.0)	0.0 (0.0)	7.8 (6.0–9.6)	15.1 (12.7–17.4)	24.8 (21.9–27.6)	27.1 (24.2–30.0)	29.2 (26.3–32.2)
Female	966	0.1 (0.01–0.3)	5.0 (3.6–6.4)	6.3 (4.8–7.9)	9.4 (7.6–11.3)	13.4 (11.2–15.5)	11.3 (9.3–13.3)	11.5 (9.5–13.5)
*HbA_1c_*								
All	1823	0.2 (0.01–0.35)	2.5 (1.8–3.2)	6.7 (5.5–7.8)	11.6 (10.2–13.1)	19.1 (17.3–20.9)	18.1 (16.3–19.9)	20.0 (18.2–21.9)
Male	872	0.2 (0.01–0.6)	0.0 (0.0)	7.8 (6.0–9.6)	14.7 (12.3–17.1)	25.6 (22.7–28.5)	26.1 (23.2–29.1)	28.9 (25.9–31.9)
Female	951	0.1 (0.01–0.3)	4.7 (3.4–6.1)	5.7 (4.2–7.2)	8.8 (7.0–10.6)	13.1 (11.0–15.3)	10.7 (8.8–12.7)	11.9 (9.8–13.9)

**Table 2:** Extrapolation of the Mitchelstown findings to the Irish population: numbers of individuals at high risk of developing T2DM by each diabetes risk score according to gender and age group.

Gender and age group	Irish population	Wilson	Balkau	FINDRISC	Schulze	Kahn Enhanced	KahnBasic	Griffin
Male								
45–54	280,297	1,205	0	9,810	21,022	52,976	52,976	46,810
55–64	217,421	1,739	0	19,350	31,570	68,922	60,008	64,139
65–74	136,399	0	0	13,913	35,191	34,509	33,691	60,152
Total	634,117	2,945	0	43,074	87,783	156,408	146,675	171,101
Female								
45–54	283,278	0	9,065	12,464	13,597	9,065	18,130	9,065
55–64	216,791	434	12,574	14,959	19,728	34,687	35,987	23,847
65–74	141,208	0	7,060	9,885	22,734	12,709	19,204	32,619
Total	641,277	434	28,699	37,307	56,060	56,460	73,321	65,531
Overall total	1,275,394	3,378	28,699	80,381	143,843	212,868	219,996	236,632

### Cardiometabolic Risk Profiles According to Diabetes Risk Scores

**Figure 2:**
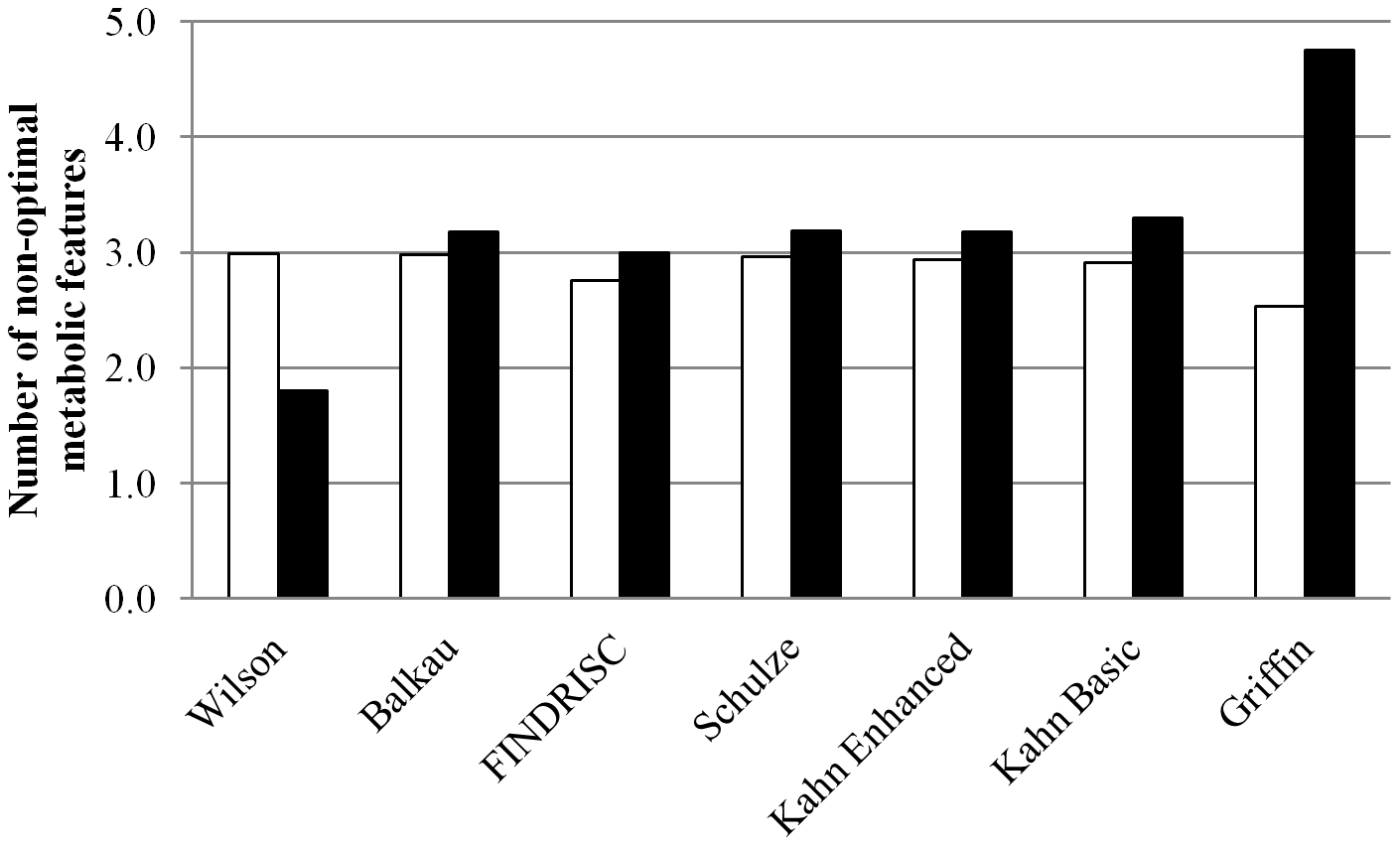
Number of non-optimal metabolic features among subjects according to each diabetes risk score. Significant differences between individuals classified as at high risk of developing T2DM (black bars) and those classified as not being at high risk (white bars) were observed for the Griffin (*P*<0.001) and Kahn Basic risk scores (*P*<0.005).

Greater numbers of non-optimal metabolic features were generally observed among individuals classified as at high risk of developing T2DM, with the exception of the Wilson score (Figure 2). Significant differences between subjects were observed for the Griffin (*P*<0.001) and Kahn Basic risk scores (*P*<0.005). Closer examination of individual inflammatory profiles, clinical characteristics, anthropometric measurements and markers of lipid and glucose homeostasis according to diabetes risk classification are presented in Table 3. Only the Griffin risk score identified a range of significant differences. Compared to their low risk counterparts individuals at high risk of developing T2DM had larger waist circumference, higher BMI, were more hypertensive, which may be expected as these variables are included in the risk score. Interestingly these subjects also displayed a more pro-inflammatory, pro-thrombotic, dysglycaemic and more insulin resistant metabolic phenotype.

**Table 3:** Cardiometabolic profiles according to each diabetes risk score^a^.

		Wilson	Balkau	FINDRISC	Schulze	Kahn Enhanced	Kahn Basic	Griffin
Age (years)	At risk	57.3±5.5^a^	59.5±6.4	60.4±5.5	60.4±5.5	59.8±5.6	59.9±5.5	62.3±5.1^c^
	Not at risk	59.7±5.2	59.7±5.5	59.6±5.4	59.6±5.4	59.6±5.5	59.6±5.5	59.0±5.4
BMI (kg/m^2^)	At risk	28.5±3.8	28.8±4.6	28.7±4.6	28.7±4.7	28.7±4.7	28.8±4.7	32.3±4.1^c^
	Not at risk	28.4±4.6	28.4±4.5	28.4±4.1	28.4±4.2	28.3±4.1	28.3±4.4	27.4±4.2
Waist circumference (cm)	At risk	97.6±13.1	97.7±13.1	97.0±13.3	97.2±13.3	96.9±13.4	97.5±13.2	107.2±10.18^c^
	Not at risk	96.3±13.9	96.2±14.6	96.2±11.2	96.1±12.3	96.1±11.9	96.0±12.9	93.5±12.14
SBP (mmHg)	At risk	12317	124±17^b^	129±17	130±18	129±17	129±17	135±17^c^
	Not at risk	130±16	130±13	129±17	129±17	130±16	129±16	128±17
DBP (mmHg)	At risk	74±10^b^	78±10^b^	80±11	80±11	80±10	80±10	82±10^c^
	Not at risk	80±6	80±9	80±10	80±10	80±9	80±10	80±10
FPG (mmol/L)	At risk	4.60±0.57	5.07±0.68	4.96±0.57	4.98±0.62	5.03±0.57	5.03±0.61	5.23±0.67^c^
	Not at risk	4.98±0.53	4.98±0.56	4.98±0.54	4.98±0.58	4.97±0.56	4.97±0.56	4.93±0.52
HbA1c (%)	At risk	5.40±0.49	5.81±0.37^b^	5.69±0.37	5.73±0.35	5.72±0.38	5.92±0.38	5.85±0.41^c^
	Not at risk	5.71±0.35	5.71±0.35	5.71±0.34	5.70±0.35	5.70±0.34	5.71±0.34	5.67±0.32
Insulin (μIU/ml)	At risk	12.09±13.3	12.09±8.98	10.48±9.07	12.16±11.4	11.56±9.88	11.71±10.50	16.21±12.10^c^
	Not at risk	10.80±8.98	10.77±9.56	10.83±8.01	10.62±8.61	10.64±8.78	10.60±8.61	9.46±7.50
HOMA-IR	At risk	2.34±2.26	2.87±2.60	2.40±2.22	2.79±2.39	2.68±2.62	2.69±2.65	3.84±3.11^c^
	Not at risk	2.47±2.27	2.46±2.24	2.47±2.40	2.42±2.15	2.41±2.16	2.41±2.15	2.13±1.86
HDL-C (mmol/L)	At risk	1.52±0.58	1.41±0.37	1.49±0.37	1.45±0.37	1.46±0.37	1.43±0.37	1.28±0.32^c^
	Not at risk	1.46±0.37	1.46±0.36	1.46±0.37	1.47±0.37	1.46±0.37	1.47±0.37	1.51±0.37
TAG (mmol/L)	At risk	1.45±0.81	1.52±0.82	1.40±0.81	1.44±0.96	1.49±0.98^b^	1.48±0.95^b^	1.69±0.98^c^
	Not at risk	1.39±1.15	1.38±0.78	1.39±0.83	1.38±0.79	1.36±0.77	1.36±0.78	1.31±0.74
CRP (ng/ml)	At risk	1.56±3.67	2.55±3.65	2.17±3.72	2.18±3.75	2.43±3.70	2.36±3.74	2.78±4.23^c^
	Not at risk	2.34±1.15	2.34±4.27	2.35±3.02	2.36±3.00	3.32±3.56	2.34±3.36	2.21±3.46
C3 (mg/dl)	At risk	138.9±24.4	143.4±30.6	138.4±24.3	138.4±24.7^b^	139.8±24.3^c^	139.0±25^c^	141.2±23.4^c^
	Not at risk	135.4±21.2	135.2±24.2	135.2±26.7	135.0±22.7	134.5±24.6	134.6±24.2	133.9±24.4
TNF-a(pg/ml)	At risk	5.27±2.49	6.09±2.50	6.48±2.55	6.50±2.45	6.50±2.51	6.53±2.71	6.94±2.88^c^
	Not at risk	6.31±1.72	6.31±1.80	6.29±2.49	6.26±2.77	6.25±2.41	6.25±2.43	6.13±2.34
IL- 6 (pg/ml)	At risk	2.40±4.96	2.81±4.98	3.64±4.83	3.05±5.05	3.24±5.33	3.38±6.01	3.90±4.88^c^
	Not at risk	2.90±1.55	2.90±3.51	2.84±3.21	2.88±4.94	2.82±4.85	2.79±4.67	2.62±4.85
Adiponectin (ng/ml)	At risk	6.53±5.44	6.55±3.95	5.94±3.96	5.71±4.03	5.98±4.58	5.71±3.96	4.48±4.10^c^
	Not at risk	5.81±3.95	5.80±4.08	5.81±4.09	5.83±3.95	5.78±3.80	5.84±3.93	6.18±3.04
Leptin (ng/ml)	At risk	1.61±1.28	2.99±6.34	3.01±3.44	2.98±3.57	2.76±3.58	2.92±4.09	3.75±4.10
	Not at risk	3.10±1.20	3.10±4.46	3.09±3.22	3.10±3.43	3.17±3.01	3.13±3.03	2.91±3.93
PAI-1 (ng/ml)	At risk	25.92±12.71	29.27±12.70	27.64±12.75	27.79±12.75	28.01±12.79	27.61±12.73	29.92±13.26^c^
	Not at risk	27.22±11.14	27.17±12.61	27.18±11.95	27.14±12.30	27.03±12.25	27.12±12.56	26.53±12.42

a Values are presented as means ± SD.

b Represents *P* value <0.05.

c Represents *P* value <0.001.

### Comparison between Diabetes Risk Scores

**Figure 3:**
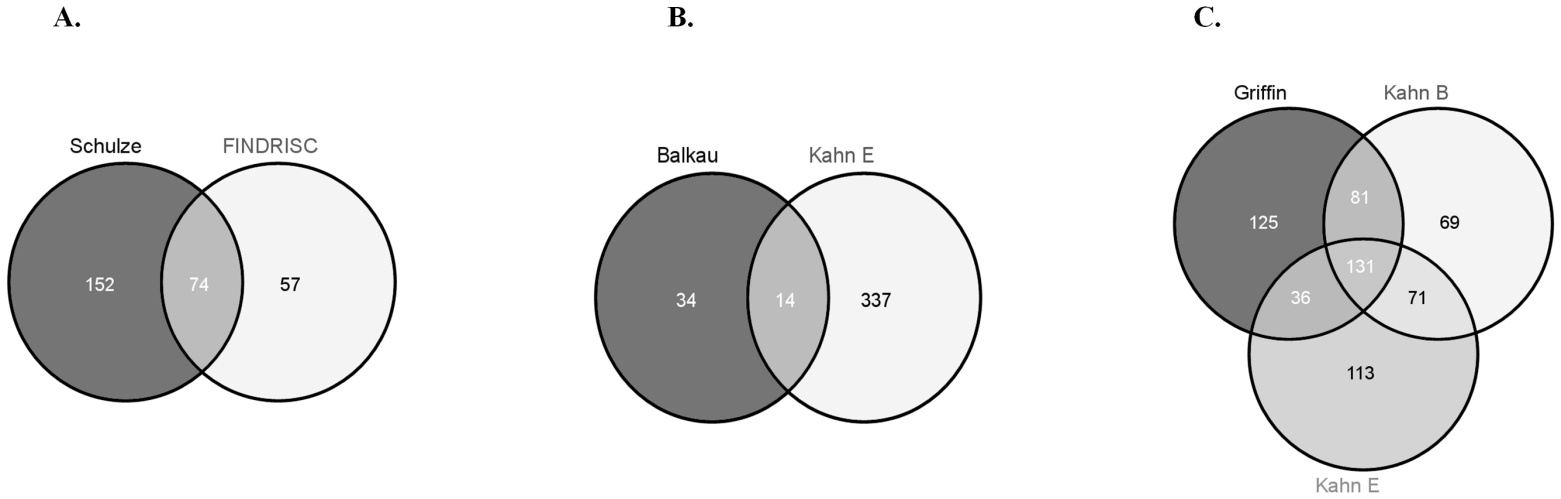
Agreement regards whether the same individuals were classified as at risk according to the different scores was examined. Good agreement was achieved between the two risk scores based on lifestyle factors (Figure 3A) with 56.5% of the subjects classified at risk by FINDRISC being simultaneously classified as at risk by the Schulze risk score. Lower concordance (29.2%) was observed when the three clinical based risk scores were compared (Figure 3B). Agreement was even lower (20.9%) when the three risk scores which led to the greatest prevalence were compared (Figure 3C), suggesting that these risk scores do not classify the same people as being at risk.

We examined whether the same individuals were classified as high risk according to the different scores. Despite moderate to strong positive correlations between scores (Table S4), indicating similar ordering of subjects, agreement levels between risk score classifications were low. Comparing the two lifestyle factor based risk scores (FINDRISC and Schulze) revealed some degree of concordance (Figure 3A). These scores identified a total of 283 subjects at risk: n = 226 (Schulze) and n = 131 (FINDRISC). Of the 131 participants at risk according to FINDRISC 74 (56.5%) of these were simultaneously classified as at risk by Schulze (Cohen’s kappa 0.37, *p*<0.001). Comparison of the clinical risk scores (Balkau, Wilson and Kahn Enhanced) was disappointing (Figure 3B), even after exclusion of the Wilson score, as only 14 (29.2%) of the 48 subjects classified as at risk according to Balkau were similarly classified by Kahn Enhanced which identified 351 subjects as being at risk. Comparison of the risk scores which led to the greatest prevalence (Kahn Basic and Enhanced, Griffin) revealed that only 131 subjects (20.9%) were simultaneously classified as high risk according to all three of these scores (Figure 3C).

## Discussion

Several diabetes risk scores have been developed as screening tools to identify individuals either with undiagnosed T2DM and/or at high risk of developing T2DM. However it is not clear which risk scores are the best or who should be screened using such scores. Comparative data on the performance of a range of diabetes risk scores in a given population is limited. Therefore the aims of this study were to compare the results of diabetes risk assessment tools based on a range of anthropometric, clinical, biological, family history and/or lifestyle data in estimating risk of developing T2DM in a middle-aged Irish population and to characterise their cardiometabolic profiles according to each tool.

Estimates of at risk subjects in the Mitchelstown cohort varied considerably according to the risk score used, with higher proportions of high risk males identified. Extrapolation of these risk estimates to the Irish population revealed that between 3,378 to 236,632 adults are at high risk of developing T2DM. Similar but slightly lower numbers of at risk males and females were identified when HbA_1c_ rather than FPG was used to exclude diabetic subjects. The inclusion, or indeed exclusion, of certain factors, differential weighting of each variable, variation in high risk thresholds and differences in populations used to develop these scores contributed to the wide range of risk estimates obtained. While the risk scores were based on a range of variables, some factors were shared between scores. The constellation of hypertension, obesity, dysglycaemia and dyslipidaemia characterise the MetS which is associated with increased T2DM risk. Thus it was expected that these phenotypes would feature in diabetes risk scores. Only hypertension and a measure of obesity or adiposity were included in all scores. Most scores included personal or family history and some included biological parameters such as lipids and FPG. Modifiable risk factors including physical activity and moderate alcohol consumption are associated with reduced T2DM risk [27], [28], whereas smoking is related to increased risk [29]. Smoking was included in the Balkau, Schulze, Kahn Basic and Griffin scores. Alcohol was examined in the Schulze score and the Kahn Enhanced score. Diet is major modifiable risk factor associated with diabetes risk [30]–[33]. Only the FINDRISC and Schulze scores took diet and physical activity into account.

All of the risk scores, except for Kahn *et al.*, [13] and Wilson *et al.*, [16], were developed in European populations. One might expect these scores to be applicable to an Irish population. However increasing evidence suggests that not only can risk scores not be generalised from one country to another but that risk scores developed and used in the same country produce conflicting results [34]–[36]. The lowest risk estimates (0.3–2.6%) were obtained from the Wilson and Balkau scores, which are based on biological and clinical parameters, respectively. It should be noted that despite FPG being significantly predictive for diabetes in the Balkau study [11], it was not included in the risk score. Importantly age was not included in either score. Age is highly correlated with adiposity, hypertension and glucose concentrations. Waist circumference and hypertension appear in the Balkau risk score, but the lack of both age and FPG in the model may partly account for the low estimates. Also noteworthy is the finding that only women were identified as being at risk according to the Balkau score, which may introduce a gender bias to analysis based on this score. Furthermore the Framingham Offspring Study, from which the Wilson risk score was developed, was initiated more than 20 years ago. Thus it could be argued that their data may not be an accurate reflection of current diabetes trends with respect to lifestyle behaviour.

The highest prevalence estimates (18.9–20%) were obtained for the Griffin and Kahn scores (Basic and Enhanced), which all include age. Alcohol intake was only included in the Kahn Enhanced risk score, which pooled non-drinkers and former drinkers into a single group, thereby not taking the U-shaped association between alcohol intake and T2DM risk into account. The Griffin score was based on a Caucasian UK population aged 40–79 years, whereas the Kahn US population (45–64 years of age) included 22.8% black participants. Different scoring was applied according to race, which would not impact on our findings, but the accuracy of the Kahn scores in predicting diabetes risk for people older than 64 years of age has not been confirmed. Indeed comparison of risk estimates from each score according to age group revealed lowest risk in the 45–54 year old age group for all scores. It is thought that over the next 25 years the greatest increase in T2DM in developed countries will be observed in the over 65 year old age group [37]. In keeping with this greatest risk was identified in the 65–74 year olds by all scores, except for the Balkau and both Kahn risk scores which detected greatest risk in the 55–64 year olds. The lack of age in the Balkau score and younger population used to derive the Kahn scores may explain these discrepancies. Despite the above issues the sequential application of the Kahn Basic and Enhanced scores may hold some value in identification of at risk subjects. A recent prospective study of a large elderly UK population demonstrated that a two-stage approach, consisting of an initial simple clinical assessment to identify individuals who would benefit from further routine blood testing, represents an easy and cost-effective way of detecting high risk individuals [38].

Lifestyle and pharmacological interventions can delay or prevent the development of T2DM [6], [7], [9], [39]. Lifestyle modification, in particular weight loss and physical activity, can significantly reduce diabetes risk [6], [7], [9] and can be even more effective than medication [6], [39]. Although modifiable risk factors may be more informative to include in risk scores, with a view to risk reduction, most of the current risk scores are predominantly based on non-modifiable risk factors. Only the FINDRISC and Schulze scores include diet and physical activity. The FINDRISC score is the most widely used diabetes risk score which has also been successfully implemented in prevention programs [40]. Interestingly both of these scores generated risk estimates consistent with recent 10 year predictions for the Irish population [18]. Higher risk estimates were obtained for the Schulze score, which additionally includes moderate alcohol consumption, smoking behaviour and dietary consumption of red meat, wholegrain and coffee which are associated with diabetes risk [30]–[33]. Comparison of these scores demonstrates that over half of the FINDRISC at risk subjects were similarly classified by the Schulze score. This may be expected for risk scores which share the same variables. However comparison of the three clinical risk scores (Balkau, Wilson and Kahn Enhanced) revealed much lower concordance, even after exclusion of the Wilson score. Agreement between the risk scores which led to the greatest prevalence (Griffin, Kahn Basic and Enhanced) was also poor, suggesting that different risk scores identify different individuals to be at risk.

While the predictive ability of these diabetes risk scores cannot be assessed at present the planned longitudinal follow-up of the Mitchelstown cohort will enable their predictive and discriminative value to be ascertained. Nevertheless we examined the predictive value of these scores in the Cork and Kerry Phase I Study, which was initiated in 1998 (n = 1018) and re-screened in 2008 (n = 359) [41], [42]. Risk estimates were consistent with the current work, with the exception of the Schulze score which estimated the greatest risk (Table S6). Correct classification of the new T2DM cases identified in the re-screen varied considerably across scores. The Kahn Enhanced score achieved the best predictive value. The high proportion of subjects identified as at risk but who did not develop T2DM over the 10 year follow-up underscores the poor sensitivity and positive predictive value of existing diabetes risk scores.

Limited comparisons of diabetes risk scores in combination with cardiometabolic profiling exist. A Swiss comparative study examining the same diabetes risk scores, except for the Schulze score, also reported wide variation in predicted risk estimates, with the lowest risk identified by the Wilson and Balkau scores and the greatest risk by the FINDRISC and Griffin scores [36]. No dietary or second-degree family history of diabetes data were available for this study, thus the authors adapted the FINDRISC score to account for this which might have impacted on their findings. No cardiometabolic profiling was undertaken. Furthermore the relatively low participation rate (41%) may limit the applicability of their findings to the general population. Mann *et al.*, analysed the validity of 3 diabetes risk score models in predicting risk in a multi-ethnic cohort [35]. While each model maintained high discriminative ability, each required recalibration when applied to a multi-ethnic cohort. While ethnicity was not detailed, race is not an issue in the current study as there were no non-white participants in the Mitchelstown cohort. Given the relationship between inflammation and insulin resistance it is reasonable to hypothesize that systemic low-grade inflammation may also contribute to T2DM risk. Elevated concentrations of CRP, IL-6 and PAI-1 have been associated with increased risk of incident T2DM [43]–[46]. Importantly we examined cardiometabolic profiles including a range of inflammatory markers in the current work. In contrast to a previous report that the FINDRISC score can identify undetected abnormal glucose tolerance and metabolic syndrome [47], only the Griffin risk score clearly differentiated the high risk subjects who displayed a more obese and hypertensive profile, as expected as these factors are included in the risk score. Of note these high risk subjects also displayed a more pro-inflammatory, pro-thrombotic, dysglycaemic and more insulin resistant metabolic profile compared to their not at risk counterparts. Our findings suggest that the Griffin score, which also identified the greatest number of high risk subjects, may be the most clinically useful in terms of identifying individuals at greatest risk of T2DM and related metabolic perturbations.

Our study has several strengths including a high participation rate (67%), inclusion of questionnaires to assess dietary and lifestyle behaviours, detailed family and medical histories, extensive biochemical profiling and collection of anthropometric measurements which allowed us to generate diabetes risk scores using a range of anthropometric, clinical, biological and/or lifestyle factors and also to compare cardiometabolic profiles of at risk individuals according to each score which has not been achieved in comparative studies to date [35], [36]. Notwithstanding these strengths some limitations can be identified. Overall gender distribution very closely matched that of the Irish population in the 45–74 year age group (Table S5), however when age category was taken into consideration the Mitchelstown cohort had less younger (45–54) and more older (55–64) male and female participants than the corresponding Irish population, which might be explained by the fact that our study aimed to primarily recruit middle-aged subjects by randomly selecting participants in the 50–69 year age group. Considering the ageing Irish population and that the over 65 year old age group represent the highest risk group [37], our results may underestimate the true prevalence of Irish adults at risk for developing T2DM. The range of at risk estimates observed in our study may also impact on statistical power. Specifically the small numbers of subjects identified by the Balkau and Wilson scores may significantly reduce, and conversely the greater numbers of subjects identified by the Kahn and Griffin scores may significantly increase statistical power, and thus likelihood to detect significant findings.

In conclusion, we demonstrate wide variation in the estimates of middle-aged people at risk for developing T2DM according to each risk score used suggesting that these risk assessment tools require validation for each population under consideration. This data highlights the need to develop an Irish diabetes risk score which at an individual level (if designed for self-assessment by a lay person) could promote self awareness of risk factors and modifiable risk behaviours and at a national level (if designed for a use by a health professional) could identify diabetes hot spots for targeted public health interventions. Early identification of high risk individuals could allow earlier diagnosis and personalised and/or public health targeted interventions, thus attenuating the development of diabetes and associated cardiometabolic complications. For example, risk stratification using a two step approach consisting of a preliminary assessment based on a risk score followed by more in depth biological and clinical measurements may offer a more cost effective strategy to identify high risk individuals.

## Supporting Information

Table S1 Characteristics of each study used to develop diabetes risk scores.
(DOCX)

Table S2 Characteristics of each model for predicting incident diabetes risk.
(DOCX)

Table S3 Characteristics of the non-diabetic Mitchelstown cohort assessed by the diabetes risk scoring tools.
(DOCX)

Table S4 Spearman correlation coefficients between risk scores.
(DOCX)

Table S5 Comparison of gender and age distributions between the Mitchelstown cohort and the 2011 Irish population.
(DOCX)

Table S6 Comparison of diabetes risk estimates and classification of incident cases in the Cork and Kerry Phase I studies (1998 and 2008).
(DOCX)
