# Supplementary material for: Comparison of Diabetes Risk Score Estimates and Cardiometabolic Risk Profiles in a Middle-Aged Irish Population
Source: PLoS One. 2013 Nov 13;8(11):e78950. doi: 10.1371/journal.pone.0078950 (PMC3827294; doi:10.1371/journal.pone.0078950)
Supplement: Table S1 — Characteristics of each study used to develop diabetes risk scores. (DOCX) [file pone.0078950.s001.docx]

**Table S1** *Characteristics of each study used to develop diabetes risk scores*

|  | Wilson | Balkau | FINDRISC | Schulze | Kahn (enhanced) | Kahn  (basic) | Griffin |
| --- | --- | --- | --- | --- | --- | --- | --- |
| Study name | Framingham Offspring study | Data on Epidemiology of Insulin Resistant syndrome (DESIR) | Finnish Diabetes Risk Score  FINDRISC | German Diabetes Risk Score (EPIC-Potsdam) | Atherosclerosis Risk in Communities (ARIC) | Atherosclerosis Risk in Communities (ARIC) | Ely and Wessex Study |
| Country | USA | France | Finland | Germany | USA | USA | England |
| Subject numbers | 3,140 | 3,817 | 4,435 | 25,167 | 12,729 | 12,729 | 1,077 |
| Subject age (years) | 54 (SD 9.8) | 30-65 | 35-64 | 35-65 | 45-64 | 45-64 | 40-64 |
| Follow-up (years) | 8 | 9 | 5-10 | 7 | 10 | 10 | 0 ^a^ |
| Cut-off used to define high risk | 33% | 30% | Score of 12 | Score of 750 | 46.1% | 33% | 37% |
| Area under ROC | 0.85 | 0.71 for men  0.83 for women | 0.86 | 0.84 | 0.79 | 0.71 | 0.80 |

^a^ cross-sectional study, diabetes risk score was used for incident diabetes risk estimation in 3 year follow-up of EPIC-Norfolk cohort [[43](#_ENREF_43)].
