# Supplementary material for: Comparison of Diabetes Risk Score Estimates and Cardiometabolic Risk Profiles in a Middle-Aged Irish Population
Source: PLoS One. 2013 Nov 13;8(11):e78950. doi: 10.1371/journal.pone.0078950 (PMC3827294; doi:10.1371/journal.pone.0078950)
Supplement: Table S2 — Characteristics of each model for predicting incident diabetes risk. (DOCX) [file pone.0078950.s002.docx]

**Table S2** *Characteristics of each model for predicting incident diabetes risk*

|  | Wilson | Balkau | FINDRISC | Schulze | Kahn (enhanced) | Kahn (basic) | Griffin |
| --- | --- | --- | --- | --- | --- | --- | --- |
| Age |  |  | X | X | X | X | X |
| Gender |  |  |  |  |  |  | X |
| Lifestyle |  |  |  |  |  |  |  |
| Diet |  |  | X | X |  |  |  |
| Physical activity |  |  | X | X |  |  |  |
| Smoking |  | X |  | X |  | X | X |
| Alcohol intake |  |  |  | X | X |  |  |
| Genetics |  |  |  |  |  |  |  |
| Ethnicity |  |  |  |  | X | X |  |
| Family history | X | X |  |  | X | X | X |
| Personal history |  |  | X |  |  |  |  |
| Anthropometry |  |  |  |  |  |  |  |
| Waist circumference |  | X | X | X | X | X |  |
| Weight |  |  |  |  |  | X |  |
| Height |  |  |  | X | X | X |  |
| BMI | X |  | X |  |  |  | X |
| Biological and clinical |  |  |  |  |  |  |  |
| Fasting glucose | X |  | X |  | X |  |  |
| HDL-cholesterol | X |  |  |  | X |  |  |
| Triglycerides | X |  |  |  | X |  |  |
| Uric acid |  |  |  |  | X |  |  |
| Resting pulse |  |  |  |  | X | X |  |
| Hypertension | X | X | X | X | X | X | X |
| Other |  |  |  |  |  |  |  |
| Prescribed steroids |  |  |  |  |  |  | X |
