# Supplementary material for: Comparison of Diabetes Risk Score Estimates and Cardiometabolic Risk Profiles in a Middle-Aged Irish Population
Source: PLoS One. 2013 Nov 13;8(11):e78950. doi: 10.1371/journal.pone.0078950 (PMC3827294; doi:10.1371/journal.pone.0078950)
Supplement: Table S3 — Characteristics of the non-diabetic Mitchelstown cohort assessed by the diabetes risk scoring tools. (DOCX) [file pone.0078950.s003.docx]

**Table S3** *Characteristics of the non-diabetic Mitchelstown cohort assessed by the diabetes risk scoring tools* *^a^*

|  | Men  (n=896) | Women  (n=966) | P value |
| --- | --- | --- | --- |
| Age (years) | 59.6±0.18 | 59.7±0.18 | 0.659 |
| Genetics |  |  |  |
| Family history, (%) | 14.2 | 19.8 | 0.002 |
| Personal history, (FPG ≥5.55) (%) | 19.2 | 9.6 | <0.0001 |
| Personal history, (case history) (%) | 0 | 0 | 0.483 |
| Anthropometry |  |  |  |
| Waist circumference (cm) | 101.9±0.36 | 90.8±0.39 | <0.0001 |
| Weight (kg) | 86.9±0.45 | 71.3±0.43 | <0.0001 |
| Height (cm) | 173±0.2 | 160±0.18 | <0.0001 |
| BMI (kg/m^2^) | 28.9±0.14 | 27.9±0.16 | <0.0001 |
| Dietary intake |  |  |  |
| Fruit and vegetable (portions/day) | 7.11±0.19 | 7.31±0.16 | 0.431 |
| Red meat (150g portions/day) | 0.81±0.02 | 0.71±0.02 | <0.0001 |
| Wholegrain (50g portions/day) | 1.16±0.05 | 1.12±0.04 | 0.464 |
| Coffee (150g portions/day) | 1±0.06 | 1.24±0.06 | 0.006 |
| Physical activity |  |  |  |
| Low, (%) | 41.5 | 53.2 | <0.0001 |
| Moderate, (%) | 28.4 | 31.7 | 0.228 |
| Vigorous, (%) | 30.1 | 15.1 | <0.0001 |
| Moderate & vigorous (hr/week) | 8.74±0.68 | 2.32±0.31 | <0.0001 |
| Smoking |  |  |  |
| Never, (%) | 42.5 | 57.9 | <0.0001 |
| Former, (%) | 39.8 | 25.4 | <0.0001 |
| Current, (%) | 13.6 | 14.2 | 0.023 |
| Alcohol intake |  |  |  |
| Drinkers, (%) | 79.5 | 69 | <0.0001 |
| Never drinkers, (%) | 20.5 | 31 | <0.0001 |
| Moderate alcohol consumers  (10-40g/day), (%) | 17.4 | 9.6 | <0.0001 |
| Biological and clinical characteristics |  |  |  |
| Fasting glucose (mmol/L) | 5.08±0.02 | 4.89±0.02 | <0.0001 |
| HDL-cholesterol (mmol/L) | 1.29±0.01 | 1.63±0.02 | <0.0001 |
| Triglycerides (mmol/L) | 1.56±0.03 | 1.22±0.02 | <0.0001 |
| Uric acid (μmol/L) | 355.6±2.8 | 280.2±2.2 | <0.0001 |
| Resting pulse (bpm) | 70.1±0.41 | 72.6±0.36 | <0.0001 |
| Hypertension, (%) | 52 | 49 | 0.215 |
| Prescribed steroids, (%) | 2.1 | 1.8 | 0.572 |

^a^ Values expressed as means ± SEM or % where appropriate.
