# Supplementary material for: Comparison of Diabetes Risk Score Estimates and Cardiometabolic Risk Profiles in a Middle-Aged Irish Population
Source: PLoS One. 2013 Nov 13;8(11):e78950. doi: 10.1371/journal.pone.0078950 (PMC3827294; doi:10.1371/journal.pone.0078950)
Supplement: Table S4 — Spearman correlation coefficients between risk scores. (DOCX) [file pone.0078950.s004.docx]

**Table S4** *Spearman correlation coefficients between risk scores*

|  | Wilson | Balkau | FINDRISC | Schulze | Kahn Enhanced | Kahn  Basic | Griffin |
| --- | --- | --- | --- | --- | --- | --- | --- |
| Wilson | 1.000 | 0.53 | 0.56 | 0.56 | 0.81 | 0.64 | 0.62 |
| Balkau |  | 1.00 | 0.75 | 0.75 | 0.68 | 0.83 | 0.65 |
| FINDRISC |  |  | 1.00 | 0.74 | 0.69 | 0.79 | 0.70 |
| Schulze |  |  |  | 1.00 | 0.75 | 0.84 | 0.74 |
| Kahn Enhanced |  |  |  |  | 1.00 | 0.83 | 0.68 |
| Kahn Basic |  |  |  |  |  | 1.00 | 0.77 |
| Griffin |  |  |  |  |  |  | 1.00 |
