# Supplementary material for: Comparison of Diabetes Risk Score Estimates and Cardiometabolic Risk Profiles in a Middle-Aged Irish Population
Source: PLoS One. 2013 Nov 13;8(11):e78950. doi: 10.1371/journal.pone.0078950 (PMC3827294; doi:10.1371/journal.pone.0078950)
Supplement: Table S5 — Comparison of gender and age distributions between the Mitchelstown cohort and the 2011 Irish population. (DOCX) [file pone.0078950.s005.docx]

**Table S5** *Comparison of gender and age distributions between the Mitchelstown cohort and the 2011 Irish population*

| Age group  (years) | Mitchelstown  (n=2,047) | Irish population  (n=1,347,707 in 45-74 age group) |
| --- | --- | --- |
| All |  |  |
| 45-54 | 25.0 | 43.0 |
| 55-64 | 54.0 | 34.4 |
| 65-74 | 21.0 | 22.6 |
| Male (%) | 1008 (49.2) | 49.7 |
| 45-54 | 24.8 | 43.0 |
| 55-64 | 54.1 | 34.6 |
| 65-74 | 21.1 | 22.4 |
| Female (%) | 1039 (50.8) | 50.3 |
| 45-54 | 25.2 | 43.0 |
| 55-64 | 54.0 | 34.1 |
| 65-74 | 20.8 | 22.9 |
