# Supplementary material for: Comparison of Diabetes Risk Score Estimates and Cardiometabolic Risk Profiles in a Middle-Aged Irish Population
Source: PLoS One. 2013 Nov 13;8(11):e78950. doi: 10.1371/journal.pone.0078950 (PMC3827294; doi:10.1371/journal.pone.0078950)
Supplement: Table S6 — Comparison of diabetes risk estimates and classification of incident cases in the Cork and Kerry Phase I studies (1998 and 2008). (DOCX) [file pone.0078950.s006.docx]

**Table S6** *Comparison of diabetes risk estimates and classification of incident cases in the Cork and Kerry Phase I studies (1998 and 2008)*

| Diabetes Risk Score | Proportion of high risk subjects  (95% CI) | Correct classification of incident T2DM (%) | Proportion of high risk subjects who did not develop T2DM |
| --- | --- | --- | --- |
| Wilson | 2.6 (1.6-3.6) | 6.7 | 96.0 |
| Balkau | 3.0 (1.9-4.1) | 13.3 | 93.1 |
| FINDRISC | 4.0 (2.7-5.2) | 0 | 100 |
| Schulze | 22.2 (19.6-24.9) | 53.3 | 96.3 |
| Kahn Enhanced | 19.2 (16.8-21.9) | 80.0 | 93.5 |
| Kahn Basic | 19.3 (16.9-21.9) | 6.7 | 99.5 |
| Griffin | 20.6 (18.0-23.2) | 40.0 | 96.9 |
